# More than just a gut feeling: constraint-based genome-scale metabolic models for predicting functions of human intestinal microbes

**DOI:** 10.1186/s40168-017-0299-x

**Published:** 2017-07-14

**Authors:** Kees C. H. van der Ark, Ruben G. A. van Heck, Vitor A. P. Martins Dos Santos, Clara Belzer, Willem M. de Vos

**Affiliations:** 1 Laboratory of Microbiology, Wageningen University, Stippeneng 4, 6708 WE Wageningen, The Netherlands; 2 Laboratory of Systems and Synthetic Biology, Wageningen University, Stippeneng 4, 6708 WE Wageningen, The Netherlands; 3 LifeGlimmer GmbH, Markelstrasse 38, 12163 Berlin, Germany; 4 RPU Immunobiology, Department of Bacteriology and Immunology, University of Helsinki, Haartmanikatu 4, 002940 Helsinki, Finland

**Keywords:** Microbiome, Microbiota, Genome-scale metabolic model, Culturing, Minimal media, Phenotype prediction, Interspecies interactions

## Abstract

The human gut is colonized with a myriad of microbes, with substantial interpersonal variation. This complex ecosystem is an integral part of the gastrointestinal tract and plays a major role in the maintenance of homeostasis. Its dysfunction has been correlated to a wide array of diseases, but the understanding of causal mechanisms is hampered by the limited amount of cultured microbes, poor understanding of phenotypes, and the limited knowledge about interspecies interactions. Genome-scale metabolic models (GEMs) have been used in many different fields, ranging from metabolic engineering to the prediction of interspecies interactions. We provide showcase examples for the application of GEMs for gut microbes and focus on (i) the prediction of minimal, synthetic, or defined media; (ii) the prediction of possible functions and phenotypes; and (iii) the prediction of interspecies interactions. All three applications are key in understanding the role of individual species in the gut ecosystem as well as the role of the microbiota as a whole. Using GEMs in the described fashions has led to designs of minimal growth media, an increased understanding of microbial phenotypes and their influence on the host immune system, and dietary interventions to improve human health. Ultimately, an increased understanding of the gut ecosystem will enable targeted interventions in gut microbial composition to restore homeostasis and appropriate host-microbe crosstalk.

## Background

### Understanding the gut microbiome

The human gut is colonized since birth with complex microbial communities, mainly consisting of bacteria with millions of unique genes that show substantial interpersonal variation in adult life [1]. This complex ecosystem—the gut microbiome—is an integral part of the gastrointestinal tract (GIT) and is intrinsically involved in the maintenance of body homeostasis. Aberrations in the microbial composition have been correlated to a wide array of diseases, ranging from obesity to diabetes, and from inflammatory bowel disease to autism [2, 3]. These correlations have spawned interest in developing strategies to improve human health by rationally steering this composition and thereby the function of the gut microbiome [4, 5]. This approach has been greatly stimulated by the success of transplantations of fecal microbiota, which showed that ‘bugs-can-beat-drugs’ in fighting recurrent *Clostridium difficile* infections [6]. However, rationally steering microbiome composition and function requires a thorough understanding of the causal mechanisms underpinning these correlations. Thus far, this understanding has been hampered by (i) the gap between the number cultured gut bacteria and sequenced gut bacteria, (ii) the poor phenotypic characterization of the majority of gut microbes, and (iii) the limited understanding of the interactions of microbes with each other as well as their host. As in other areas of research, the deployment of descriptive and predictive mathematical models has the potential to provide insights that ultimately enable to overcome these limitations. In this review, we will discuss the use of genome-scale constraint-based metabolic models for an increased understanding of the gut microbiome and its role in gut homeostasis and (dys)function.

### Genome-scale metabolic models in gut microbiota research

Genome-scale metabolic models (GEMs) are mathematical representations of the knowledge on an organism’s metabolic capacity and have been previously applied in bacterial systems for a variety of purposes, including the design of cultivation media, phenotypic characterizations, metabolic engineering, drug discovery, and to study interspecies interactions. For an overview of common GEM applications, we would like to refer to these reviews [7, 8].

Strong developments in both GEMs and gut microbiome research are bound to facilitate moving from correlation studies to gaining mechanistic insights. GEMs can integrate knowledge on the metabolism of one or more gut microbes and predict how this metabolic system functions in different niches in the gut. The gut environment includes nutrient gradients both along the length of the GIT, as well as along the mucosal gradient and villi, and have strong effects on the microbial function [9, 10]. GEMs provide a valuable framework for the integrated study of gut function as they enable the generation of testable hypotheses that can lead to novel insights into causal relationships between the gut microbiome and human health. Considerable progress in these relations has been obtained with the short chain fatty acids (SCFAs) that are produced as main bacterial metabolites in the colon, as illustrated for butyrate, an established functional compound [11, 12]. The impact of SCFAs on metabolic health has been reviewed recently [13]. In a model system, it was found that acetate is secreted by *Bifidobacterium adolescentis* L2-32, taken up by *Faecalibacterium prausnitzii* A2-165 and used to produce butyrate from sugar. This enabled the prediction of *F. prausnitzii* acetate requirements for butyrate production and how this relates to its low abundance in cases of Crohn’s disease [4], showing how an observed correlation can possibly be explained mechanistically using GEMs.

**Fig. 1:**
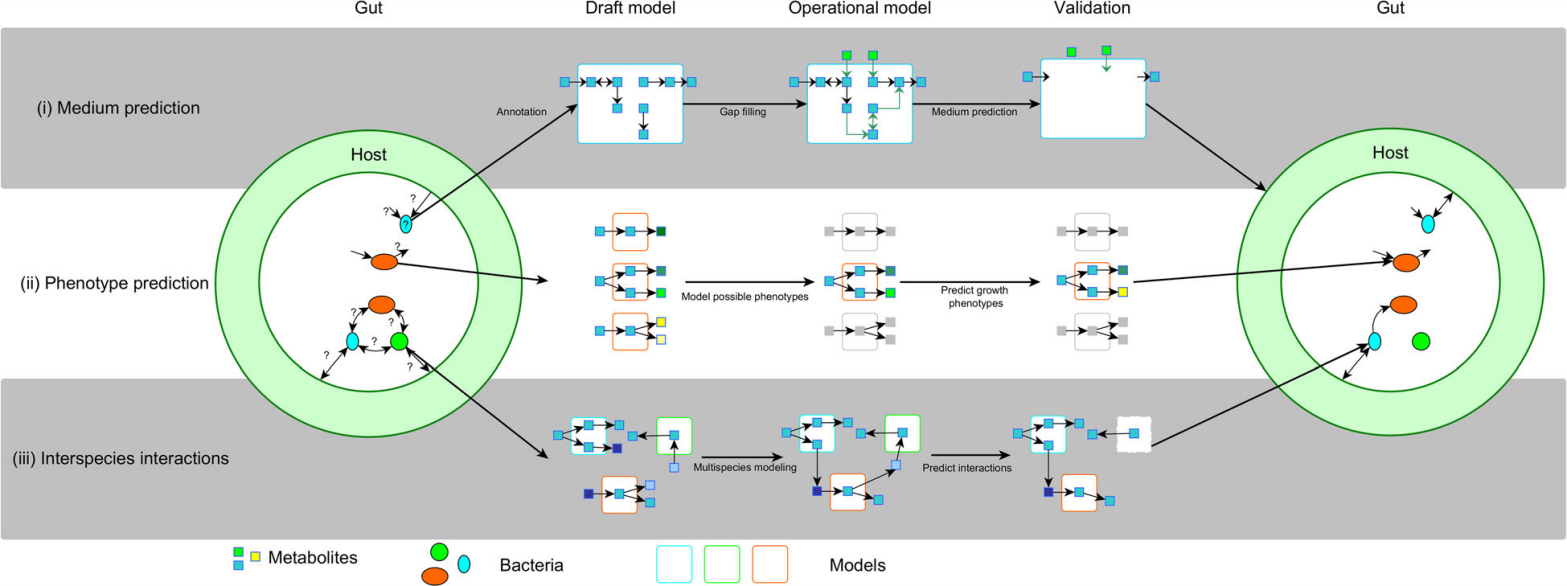
Simplified overview of the use of GEM to increase understanding of the metabolic interactions in the gut microbiome. Individual species require metabolites (*squares*) to grow. These metabolites can be predicted by GEMs, which results in medium and growth (rate) prediction (*i*, *top*). The possible solution the bacteria use to metabolize these metabolites can change under different conditions (*ii*, *middle*), which leads to altered interactions between bacteria (*iii*, *bottom*)

In the remainder of this review, we will discuss the use of GEMs in gut microbiota research and how GEMs can advance gut research toward the understanding of gut homeostasis and (dys)function. We will focus on the metabolic reactions of the microbes in the gut, on their growth, on their interactions, and on the metabolites produced. These are either primary products of microbial metabolism or breakdown products of our diets or host compounds, having a plethora of functions, ranging from SCFAs that fuel enterocytes and have specific signaling and immune functions, to vitamins and other host growth-promoting compounds [14]. Most of these metabolites cannot be easily detected in the human GIT as these are taken up by the host and processed in the liver. Since GEMs stochiometrically represent all metabolic reactions in a microbe or microbial community, such models enable to estimate the production of these transient metabolites, estimate their distributions within the global metabolic network, and provide hypotheses for the metabolic interactions among gut microbes and of those with their host. Moreover, GEMs are instrumental in optimizing growth of GIT microbes in laboratory conditions and hence are relevant for the production of biomolecules that are involved in host signaling, such as TLR ligands or specific functional proteins [15, 16]. First, we briefly describe the process of genome-scale metabolic reconstruction and its implications for network modeling. Secondly, we describe applications of GEMs for gut microbiome research that enable (i) selecting minimal and defined growth media for previously cultured as well as not yet cultured gut microbes, (ii) predicting growth and phenotypes of gut microbes and their influence on health and disease, and (iii) modeling co-cultures and multispecies interactions of gut microbes and the human host (Fig. 1).

### Genome-scale metabolic reconstruction and network modeling

The basis of GEM construction is the genome annotation of the microbe of interest since this predicts the enzymes a microbe encodes, and thereby provides a list of chemical reactions the microbe can perform. This list of chemical reactions forms the draft metabolic model, which is often far from complete [17]. Typically, there are missing reactions due to incorrect, missing, or low-quality annotations, even for well-studied organisms [18]. Moreover, our knowledge of the biochemical pathways is often insufficient, with unknown conversions still being discovered [19]. These missing reactions—also called gaps—severely limit the possibilities for GEM analyses, as parts of the metabolic network are not connected. Therefore, gap-filling algorithms are used to predict the presence of additional reactions that can be obtained from reaction databases such as KEGG [20] or Metacyc [21] and to connect disconnected parts of the network [17, 22]. Thereby, these algorithms provide hypotheses on enzymes that were missed in the genome annotation. In some cases, a corresponding gene, not initially annotated as such, is identified and the genome annotation is improved. In the remaining cases, the reactions become ‘orphan reactions,’ e.g., reactions that are thought to occur in the microbe based on existing pathways of other microbes but that have not been linked to any genes. The addition of orphan reactions might lead to erroneous model predictions, but is often essential to obtain a functioning GEM and facilitates targeted gene identification [17, 22]. Model construction and gap-filling algorithms have been extensively described elsewhere [17, 22, 23].

After gap-filling, GEMs are expected to be able to sustain in silico growth of the modeled organism. Growth is modeled as the formation of biomass in a complex organism-specific reaction involving a large number of biomass precursors such as DNA, RNA, proteins, lipids, ATP, NADPH, and various small molecules. The use of biomass precursors in bacterial GEMs has recently been thoroughly explored resulting in a shortlist of universally essential, as well as organism-specific biomass precursors [24]. If all of these precursors can be formed in the right ratios, the GEM predicts that growth is possible. The most common way to predict growth phenotypes is through flux balance analysis (FBA) [25]. FBA determines an optimal flux distribution for the production of biomass components while adhering to several types of constraints: (i) mass-balance constraints; the production and consumption of intracellular metabolites cancels out, (ii) thermodynamic feasibility constraints; reactions can only operate in thermodynamically feasible directions, and (iii) capacity constraints; fluxes through reactions are bounded to biologically feasible ranges. Capacity constraints are also used to define the medium conditions by directly defining which metabolites can be imported. Thereby, GEMs can be easily modified to simulate growth phenotypes in a wide range of different experimental conditions.

GEMs are typically evaluated by comparing predicted growth phenotypes for both wild type and mutant strains to the available experimental data. This experimental data usually consists of growth measurements for a large number of media containing different carbon, nitrogen, phosphorus, and sulphur sources. For the comparison, both the experimental data and the GEM predictions are discretized to the two states ‘growth’ and ‘no growth.’ This binary discretization leads to two different types of inconsistencies: (i) growth predicted by the GEM but not experimentally found, and (ii) growth that is experimentally validated but not predicted by the GEM. In the first case, the GEM overestimates the microbe’s abilities, suggesting it may include reactions that the microbe cannot perform. In contrast, the other case suggests that the GEM is missing reactions. This comparison can thus be used to evaluate both the annotation and the gap-filling process that underlie the GEM construction. For example, if the removal of a single reaction from the GEM results in a large improvement of GEM predictions, this suggests that this reaction was erroneously added and should be considered for removal. This process of using experimental data to find incorrect GEM predictions and subsequently making changes to the GEM has also been combined into algorithms, such as GrowMatch [26], that will make a minimal number of changes to a GEM while maximizing its coherence to experimental data.

The established manual GEM reconstruction process ultimately results in high-quality GEMs, but is extremely time-consuming [17]. The advent of high throughput sequencing and concurrent rapid increase in available biological data warrants a faster approach, which is provided by the RAVEN toolbox [27] and the ModelSEED approach [28]. In both cases, the process of genome annotation, draft GEM construction, and gap-filling has been fully automated, although extensive manual curation remains necessary to sustain a high quality [27, 28]. This curation process has recently been streamlined for gut microbes specifically as part of the AGORA metabolic GEM resource [29]. A distinguishing feature of the AGORA GEM resource is the semi-automatic curation of ModelSEED GEMs where corrections that are manually applied to a single GEM are propagated to the GEMs of other gut microbes. This semi-automatic curation both speeds up the curation process and finally results in more uniform and higher quality GEMs.

### Use of GEMs to design defined culture media

The basis of classic microbiology is the ability to culture bacteria in a pure culture on a well-defined medium. Such a well-defined medium is required for detailed metabolic analyses, growth optimization, and finally also in a feedback loop with the GEM itself to optimize the metabolic model. Moreover, well-defined media devoid of animal-derived compounds will be needed when intestinal microbes that are therapeutically effective are to be cultured and used in therapeutic settings. An example is the recently developed medium for *Akkermansia muciniphila* that was used for a human safety study [61]. Finally, obtaining pure cultures is essential for intervention studies to investigate host-microbe interactions and to use the beneficial bacteria as potential therapeutic microbes. Pure cultures have been successfully obtained for over 1000 different gut species [30], which was recently expanded by high throughput culturing approaches [31, 32]. However, as it has been predicted that there are at least two to three times more different gut species, a significant number of gut microbes remain uncultured and inaccessible for study in isolation [33]. A number of known not yet cultured candidates have been listed in a ‘most wanted’ list [34], which highlights the need for culturing of gut microbes. Among these targets are *Oscillospira* spp. that are receiving considerable attention [35–37]. A major issue in the culturing of these microbes is the lack of suitable growth media. Growth media are often based on the ecosystem a microbe naturally occurs in, but the gut is extremely complex with many different nutrients, highly variable nutrient levels, and many interspecies interactions. Here, we first describe the challenges in the use of GEMs for the design of defined media, and then how GEMs have been successfully used for the design of defined media and how similar approaches can be used to design suitable defined media for not yet cultured bacteria.

There are three main challenges in the use of GEMs for the design of defined growth media: (i) The in silico biomass composition is an influential aspect of the GEM as it defines all metabolites required for growth [24]. The omission of even a single metabolite in this composition can prevent the GEM from predicting an essential media supplement. However, the biomass composition cannot be fully determined in silico and relies on the availability of organism-specific experimental data. As this is not available for many gut microbes, automatic model generation procedures rely on heuristics to estimate the biomass components that are required for each organism [28, 29]. We highly recommend evaluating a given biomass composition generated from automatically generated GEMs according to the guidelines recently set out in a thorough evaluation of biomass compositions [24] prior to gap-filling and media design. (ii) The gap-filling step in GEM construction typically relies on the introduction of known biochemical reactions to complement the metabolic network of the modeled microbe [22]. In particular, reactions are often added such that the GEM predicts in silico growth in a pre-defined medium, which is not directly suitable if no chemically defined medium is known for the microbe or if the microbe uses not previously characterized reactions. Hence, all gap-filling reactions should be carefully individually inspected and corresponding genes need to be identified to support the procedure. (iii) GEMs do not capture the non-linear link between concentrations of medium components and the speed with which microbes can import them. Hence, GEM-based medium design is limited to predicting which compounds need to be present and cannot be used to determine optimal concentrations.

Despite these challenges, GEMs have proven to be useful in the design of chemically defined growth media, as has been shown for the lactic acid bacterium *Lactobacillus plantarum* WCFS1 [38]. Lactic acid bacteria are important in many industrial food processes and some are marketed as probiotics [39]. Therefore, the GEMs of lactic acid bacteria are used to study their metabolic capabilities and behavior in fermentation processes [40, 41], as well as their probiotic functions [42, 43]. The GEM of *L. plantarum* WCFS1 was automatically constructed based on its genome sequence and subsequently extensively manually curated [38, 44]. The GEM was then used to predict the essentiality of 36 compounds in a chemically defined growth medium. The GEM predictions were correct for 29/36 (81%) of the compounds, but were incorrect for the vitamins folate, thiamine, and vitamin B_6_, as well as for the amino acids arginine, glutamate, isoleucine, and tryptophan. The incorrect predictions pinpointed errors in both the GEM construction process and in the experimental procedures, and also pinpointed distinct metabolic features of *L. plantarum WCFS1*, for example (i) the incomplete folate biosynthesis pathway in the GEM was in part due to a missing EC number for a correctly annotated gene, as well as no reactions in Metacyc for another EC number. (ii) The GEM lacked a complete isoleucine biosynthesis pathway, but growth was observed in the isoleucine omission experiment. This turned out to be a result of isoleucine contamination in the other amino acids. (iii) A missing reaction for thiamine biosynthesis was assigned to a gene involved in molybdopterin biosynthesis. In *Enterobacteria*, these reactions are carried out by two paralogs, but it appears that both reactions are carried out by a single enzyme in *L. plantarum* [38]. These results clearly illustrate how a GEM-driven systematic evaluation of medium compositions can increase the understanding of a microbe’s metabolism.

A GEM of a different lactic acid bacterium, *Lactococcus lactis* IL1403, was constructed and used to remove all non-essential metabolites from a rich medium in order to design a minimal medium for physiological studies [45]. This exercise in medium design not only resulted in a minimal medium but also allowed for careful comparisons between in silico predictions and experimental data to understand their differences. The GEM predicted that arginine, methionine, and valine are essential for growth, and that either glutamate or glutamine is required additionally. However, recent single amino acid omission experiments have led to the conclusion that arginine, asparagine, histidine, methionine, serine, isoleucine, leucine, and valine are essential medium components for *L. lactis*, and that glutamate and glutamine are not [46]. At first glance, this might incorrectly seem like poor performance by the GEM. However, the agreements and disagreements between predictions and experiments can be summarized in three points: (i) they agree on the essentiality of arginine, methionine, valine, and the non-essentiality of the ten amino acids not previously mentioned; (ii) they do not evaluate glutamate and glutamine in the same manner—the GEM predicts that one of them is required, whereas the experiment indicates that either one can be omitted, but that glutamine cannot be omitted if the concentration of glutamate is additionally reduced to 10% of the normal concentration; and (iii) they disagree on the essentiality of asparagine, histidine, isoleucine, leucine, and serine, but also disagree on the meaning of ‘essential.’ In the *L. lactis* IL1403 GEM, a compound was essential if its omission reduced the specific growth rate below 0.01/h. In the omission experiment, a compound was considered essential if the final OD dropped below 40% of the final OD in the rich medium. This introduces a certain level of ambiguity and, for example, if the experimental threshold would instead be at 20%, asparagine and serine would not have been considered essential.

The ability to culture pathogens and probiotics is important to study them in isolation and to determine their role in the gut microbiome. Therefore, a GEM was used to design a minimal growth medium for *Staphylococcus aureus* N315, a pathogen that frequently infects hospitalized patients [47]. The GEM predicted that several amino acids were essential, but in vivo experiments indicated otherwise. Later on, an updated GEM predicted that *S. aureus* N315 has no intrinsic auxotrophies for amino acids, but that some particular isolates do require some amino acids [48]. This discrepancy between the updated GEM and the experimental results for the isolates was explained by the repression of amino acid synthesizing genes. The repression could be relieved by progressively eliminating the amino acids from the medium, supporting the GEM prediction that *S. aureus* can indeed synthesize these amino acids. This study showed how a GEM can aid in omitting nutrients from a known defined medium.

These three case studies show that GEMs are a good starting point for designing minimal media. In fact, the ability of GEMs to design growth media was recently emphasized by the development of the Minimal Environmental TOol (MENTO) [49]. MENTO predicts the minimal medium requirements for an organism based on its GEM, and was used to study broad nutritional trends in over 2500 automatically generated ModelSEED [28] models. For three well-characterized organisms, the predictions based on the ModelSEED models were also compared to the predictions based on manually curated models. The comparison indicated that the ModelSEED models are more pessimistic growth predictors, but have a similar accuracy [49]. Nonetheless, the authors indicate that while the ModelSEED models are suitable for studying broad nutritional trends, one should be careful in interpreting results for any specific organism. A ModelSEED model thus requires manual curation before using it to predict suitable minimal growth media.

Such a manually curated ModelSEED GEM was recently used for minimal medium design for *F. prausnitzii*, a prevalent and potential beneficial gut microbe that is commonly grown on the chemically undefined YCFAG medium [50]. The automatically generated ModelSEED GEM was first manually curated such that it correctly captured the known biochemistry and physiology of *F. prausnitzii*. This curation involved changing the biomass reaction, updating reaction directionalities, adding species-specific pathways, and filling gaps. The curated GEM was then used to predict a chemically defined growth medium called CDM1. CDM1 did, however, not facilitate in vitro growth and was subsequently supplemented with additional nutrients to form an extended medium CDM2, which did facilitate in vitro growth. The researchers then used LC-MS to identify what metabolites in CDM2 are net consumed, and what metabolites are net produced. The metabolite consumption and production data was then used to improve the GEM and the corresponding genome annotation. Ultimately, the researchers were able to design a refined and chemically defined medium CDM3 that facilitated both in silico and in vitro growth, albeit that growth was still rather poor and unreliable [50].

The requirement for manual curation of ModelSEED [28] GEMs prior to media design has been substantially reduced due to the presence of 773 semi-automatically curated GEMs of relevant gut microbes [30, 51] in the AGORA GEM resource [29]. These GEMs have been curated collectively such that any issues addressed in one GEM are also directly addressed in others. Although further microbe-specific manual curation may still be required for many microbes, some AGORA GEMs may also be directly suitable for media design. As a showcase, the AGORA GEM of *Bacteroides caccae* ATCC 34185 was successfully used to design the first chemically defined medium supporting in vitro growth for this gut microbe [29].

**Fig. 2:**
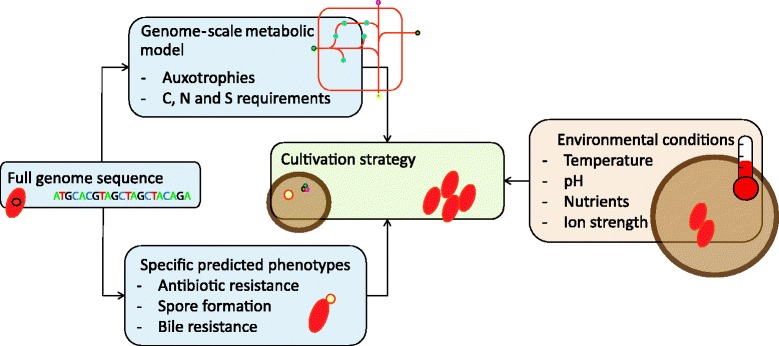
Suggested cultivation strategy. The initial cultivation strategy of a microbe can be optimized by thorough analysis of its genome and isolation conditions. The genome contains information on metabolic pathways, as represented in GEMs, that inform on auxotrophies and suitable carbon, nitrogen, and sulphur sources. In addition, the genome annotation can reveal additional considerations such as antibiotic or bile resistance, or the ability to form spores. The isolation condition of a microbe, for example the human gut, provides information on suitable environmental conditions such as temperature, pH, and ion strength

Metagenomic studies [52] and single-cell genomics [53, 54] of gut bacteria have already yielded genomes that could be used to create draft GEMs. However, the available biochemical information to turn draft GEMs into functional GEMs for uncultured bacteria is limited. To gain more insight in secreted metabolites and available nutrients in the gut, imaging mass spectrometry can be applied [55]. These uptake and secretion patterns can be incorporated into GEMs. We encourage the use of GEMs to predict minimal or defined media on which the microbes of interest can be cultured. Combined with additional ecological and genomic markers, such as temperature, antibiotic resistance, and spore formation, it should be possible to culture more bacterial species (Fig. 2). The next steps are in predicting how varying environments result in different phenotypes.

### Phenotype prediction

Most microbes have versatile and complex metabolic pathways. Often, many alternative pathways are available for the conversion of the available substrate to all biomass components. GEMs can be used to explore all possible phenotypes for a wild type or mutant strain in a given environment. In addition, GEMs can be used to interpret experimental data that is difficult to directly connect to metabolic rates, such as transcriptomics and proteomics data [56]. GEMs, which are ultimately based on genotypes, are thus a means to explore possible phenotypes in a wide range of different experimental conditions. The ability to predict how different microbial phenotypes result from different environments can ultimately have consequences for human health. For example, GEMs may be able to identify the conditions under which conditional pathogens become pathogenic [57], or, in contrast, when therapeutic bacteria or probiotics may convey their beneficial properties [42, 58].

A main challenge in the use of GEMs for the prediction of phenotypes of gut microbes is that these models are—traditionally—restricted to metabolic activities. They do not explicitly include regulation nor the synthesis of mRNAs or individual proteins. Hence, GEMs can accurately predict growth phenotypes that are related to the optimal conversion of substrates to biomass components [59], but do not directly predict the synthesis of secondary metabolites and proteins involved in crucial processes such as microbe-microbe signaling, microbe-host communication [60, 61], and inflammation [14]. Such predictions rely on the integration of ~omics data or regulatory networks, as highlighted by several of the following examples.

GEM-driven exploration of the metabolic capacities of pathogens has been explanatory for pathogenic phenotypes. For example, a GEM was used to predict virulence of *Salmonella* in a mouse model system. The GEM describes a very versatile metabolism that enables *Salmonella* to utilize 31 host nutrients, allowing it to grow fast within the host cell. The GEM predicted the pathogenicity of phenotypes and was accurate in 92% of the cases [62]. In addition, it was found that the metabolic capabilities of *Salmonella* show similarities in host dependency for growth substrates and biosynthesis to other pathogens. Like *Salmonella*, other pathogens are also capable of degrading purine nucleosides, pyrimidine nucleosides, fatty acids, glycerol, arginine, *N*-acetylglucosamine, glucose, and gluconate. Similarly, it was hypothesized that comparisons of metabolic patterns between *Pseudomonas aeruginosa* and non-pathogenic relatives could yield insight into opportunistic pathogenic phenotypes of this species [57], as has later been done successfully for *Burkholderia* species [63]. The metabolic model for the pathogenic *P. aeruginosa* also showed a versatile metabolic pattern and accounted for virulence inducing pathways, such as exopolysaccharide alginate synthesis [64].

In more recent research, highly quantitative proteomics and metabolic measurements were used to impose pH-dependent constraints on the GEM of *Enterococcus faecalis*, a human gut pathogen [65]. The pH-dependent constrained GEM accurately predicted growth rate, proton pump activity by ATPase, and a metabolic shift from mixed acid fermentation to homolactic fermentation. However, discrepancies were found between expression of lactate dehydrogenase and lactate production, which emphasized that constrains based on solely proteomic measurements are not sufficient for an accurate phenotype prediction.

Transcriptomics and proteomics experiments aim to discover what an organism is doing, but the data is often difficult to analyze because there are no one-to-one relationships between expression levels, protein quantities, enzyme activities, and fluxes [66, 67]. GEMs can aid in elucidating the metabolic activities from these data by visualizing the data on a metabolic map or by predicting metabolic fluxes [68–71]. For example, transcriptomics data of two strains of *Lactobacillus reuteri*, with potentially opposite effects on the human immune system, were analyzed by visualizing the data on two GEMs. The analysis revealed that both strains produce vitamins, essential amino acids, and mucosal binding proteins, but that they differed in their production of potential inducers of tumor necrosis factor [42]. The prediction of metabolic fluxes from ~omics data relies on the concept that, on average, gene expression levels are a proxy for fluxes. The GEM then predicts a flux distribution that matches the trends in the expression data, while accounting for mass balance, thermodynamics, and capacity constraints. Several such methods have been developed in the last few years, and have been extensively summarized and evaluated recently [71]. The evaluation did not result in a clear best-performing method, and none of the methods actually outperforms parsimonious FBA [59], which does not require any ~omics data as input. However, the evaluation conditions were limited to minimal media where the optimization of the conversion of substrates to biomass seems a suitable growth strategy. It remains to be seen how these various methods compare when microbes actively synthesize secondary metabolites in situ or in rich media.

A different approach to find out what an organism is doing, rather than what it can do, is by combining GEMs with other models, such as regulatory networks [72–74]. The regulatory networks of well-studied species such as *Escherichia coli*, *Mycobacterium tuberculosis*, and *Mycoplasma genitalium* have been elucidated and incorporated in metabolic models [75–78]. Based on these model organisms, attempts have been made to automate the incorporation of regulatory networks into GEMs [79], also especially aiming at less well-characterized species [78]. These models incorporate the influence of environmental factors on the behavior of the modeled organism, which may be extremely relevant for microbes residing in a dynamic environment such as the human gut.

These examples show how GEMs can be used to explore possible phenotypes, and to predict actual phenotypes based on ~omics data or regulatory models. However, we highlight the need for a thorough evaluation on methods for the integration of ~omics data and regulatory networks with GEMs to predict the phenotypes of gut bacteria in vitro and ultimately in vivo. This will be an important stepping-stone in predicting the role of bacteria under different gastrointestinal conditions, on which also other microbial species have a big influence.

### GEM predictions on interspecies interactions

**Fig. 3:**
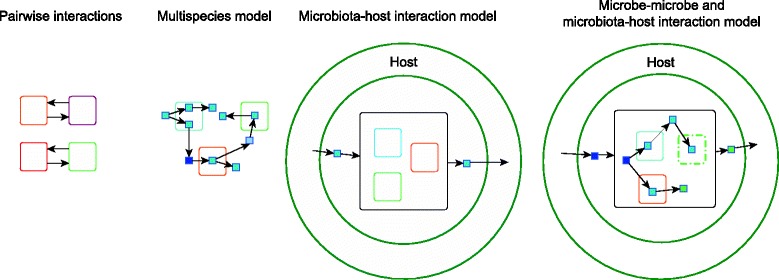
Modes of interspecies interactions as modeled before. Pairwise interactions only account for two species to share metabolites. Multispecies models allow sharing of metabolites between more than two species. Microbiota-host interaction models lump all the microbial species into one meta-model and model the interaction with the host. Microbe-microbe and microbiota-host interactions are multilevel models that take into account microbial interactions and interactions with the host

Within the gut microbiome, there are numerous microbial interactions and networks. Three types of simple multispecies interactions have been described and modeled before: mutualism, commensalism, competition, and neutralism [80–82]. GIT-colonizing microbial species often depend on each other for growth signals and substrates or compete for the metabolites, thus this ecosystem is ideal for the modeling of interspecies interactions and using interspecies interactions predictions to gain a mechanistic insight into this ecosystem [83, 84]. Interactions between microbes have been modeled on different phylogenetic levels, ranging from strains [85] to species [86, 87] and ecosystem communities [88]. The challenges in multispecies modeling are briefly described below, followed by examples of successful GEM-based multispecies modeling approaches that are also summarized in Fig. 3.

Multispecies modeling using GEMs is complicated through the aforementioned phenotype prediction challenge regarding secondary metabolites, but also by two other challenges: (i) the vast majority of GEM analysis methods rely on a steady-state assumption, but microbial interactions via signaling molecules are inherently dynamic. (ii) Flux prediction methods are based on computational optimization with regards to a single metabolic goal, usually the maximization of biomass production; a reasonable goal for an individual microbial species. However, when multiple microbes are modeled simultaneously, it is not a reasonable assumption that all work together to maximize total biomass production. The examples discussed hereafter provide a rough overview of different approaches that have been taken to minimize or circumvent these issues.

The pioneering work in GEM-based multispecies modeling was directly combining two GEMs for the mutualistic bacterium *Desulfovibrio vulgaris* and archaeon *Methanococcus maripaludis S2* into a single model with a shared extracellular environment [89]. In this ecologically relevant syntrophic relationship, *D. vulgaris* ferments lactate, and *M. maripaludis* consumes the fermentation products formate, dihydrogen, and acetate. In this work, the aforementioned issue on optimizing for biomass production was evaluated by applying distinct weights to the different types of biomass. In other words, the mathematical optimization would prioritize one type of biomass over the other in order to explore how this would affect overall flux predictions. The predicted biomass production for *D. vulgaris* was practically independent of the relative weights, whereas the *M. maripaludis* biomass production increased if it received higher weights. This is due to the sequential nature of the interaction between these bacteria, where *D. vulgaris* effectively ‘feeds’ *M. maripaludis*. However, this approach is not suitable if the community members exhibit cross-feeding or substrate competition.

A similar approach was taken to identify media that stimulate commensal or mutualistic relationships between each possible pair of seven well-known microbes [81]. This number was rapidly expanded to 118 species coupled in 6.903 pairs driven by automated curation of over a hundred GEMs [82]. The latter study not only focused on cooperation but also specifically on identifying media that induce competition between pairs of microbes. It was found that competition was generally ‘won’ by species that grew fast on versatile media, such as *E. coli*, while cooperation was more evident in *Clostridia* species that were able to degrade lignin and cellulose, which releases free sugars to other bacteria. This type of macromolecule degradation is highly important in degradation of host dietary compounds and thus directly relates to gut health.

Instead of looking into the details of the interactions between a few species, GEMs have also been used to elucidate general properties of the co-occurrence of microbes. Specifically, there are two main mechanisms driving species co-occurrence: (i) habitat filtering: microbes occupy a similar nutritional niche and compete, and (ii) species assortment: microbes have complementary metabolisms and cooperate. A recent study aimed to identify which of these two mechanisms is the driving force behind the co-occurrence of microbes in the human gut [88]. Therefore, they automatically generated 154 GEMs based on KEGG [20, 90] for gut microbes whose co-occurrences were determined based on a gut metagenome dataset containing measurements from 124 individuals. These GEMs were used to determine metabolic competition and complementarity indices between each pair of species based on network topology, thereby circumventing the need for optimization based on an ambiguous multispecies metabolic goal. As the species co-occurrence was best explained via the metabolic competition index, the authors concluded that habitat filtering is the main driving force behind species co-occurrence in the human gut. In another recent study, GEMs were used to study species co-occurrence based on 261 microbial species in 1297 communities from diverse habitats [91]. The GEMs were used to calculate both the resource competition and interaction potential within these communities based on network topology. Resource competition was significantly higher in the 1297 communities versus random assemblies, indicating that habitat filtering was again identified as the main driving force behind community composition. However, there were also 7221 sub-communities of up to 4 co-occurring species within the larger communities. Within these sub-communities, the interaction potential—defined as the difference in minimal number of metabolites required for growth between a non-interacting and a cooperating community—was significantly higher than in full communities and random assemblies.

In order to understand how gut communities form and change, it is also important to consider spatial and temporal effects. The novel modeling framework COMETS [92]—Computation of Microbial Ecosystems in Time and Space—simulates multiple GEMs on a lattice over time using dynamic FBA [93], which is based on simulating dynamics using successive steady-state optimizations. COMETS does not require any prior information on how the modeled microbes interact, but nonetheless captures interesting and non-intuitive spatiotemporal dynamics of multispecies interactions. For example, it correctly predicted that the slowest-growing microbe of a three-species ecosystem would also ultimately be the most-prevalent one, and that the growth rate of a colony with a mutualistic partner can be improved by placing a competing colony in between them. COMETS has also been used to study how robust competing and mutualistic interactions are to genetic perturbations. Specifically, it has been possible to predict the effects of gene knockouts on a synthetic community of *E. coli* and *Salmonella enterica* [94] on competition-inducing and mutualism-inducing growth media [95]. Interestingly, the community was more robust to genetic perturbations in *E. coli* under cooperative conditions, but more robust to genetic perturbations in *S. enterica* under competing conditions [95]. These results highlight that GEMs can mechanistically explain the intriguing interactions of multispecies interactions.

A conceptually similar framework is BacArena [96]. BacArena also uses a dynamic form of FBA simulations to model microbes over time, but simulates individual microbes across a 2D grid [96] rather than microbial communities on a lattice as in COMETS [92]. Of particular interest is the application of BacArena to the seven species SIHUMI community representative of a simplified human gut [97]. Initial simulations excluding glycan production in the lumen resulted in a community dominated by *E*. coli. However, as a mucus glycan gradient was imposed using diffusion on the 2D grid, the glycan-degrading *Bacteroides thetaiotaomicron* became dominant in the mucosal layer, while the lumen represented a more varied community still dominated by *E. coli*.

A multispecies interaction of particular interest is the interaction between gut microbes and their host. The host is not only an important environmental factor for gut microbes, but is also metabolically active itself. Additionally, host behavior such as diet intake has a great and reproducible influence on the microbiota composition [98]. GEMs have been created for hosts of particular interest, such as mouse [99] and human [100], and have even been trimmed down to tissue-specific GEMs, including a GEM for colon-derived tissue [101]. The human Recon 2.04 GEM was adapted to be not only tissue specific but context specific as well. Transcriptome data obtained from inflamed mucosal tissue in inflammatory bowel disease (IBD) data was used to generate new GEMs [102]. Subsequent combination of this data with bacterial expression data showed uncoupling of host-microbe metabolic interactions in IBD patients. The mouse GEM [99] was recently used to study how different diets and the presence of the gut microbe *B. thetaiotaomicron* affect its metabolism [50]. A *B. thetaiotaomicron* model was constructed using ModelSEED [28] and, after manual curation, was linked to the mouse GEM via a shared lumen compartment. Although a single microbe is not directly representative of the gut community, the combined GEM mechanistically explained how both organisms benefit from the mutualism, correctly predicted how the interaction affects biofluid metabolome composition, and even described how gut microbes can rescue hosts with lethal gene deletions [50].

Host-microbe interactions have also been modeled using a single ‘supra-organism model’ [84] to represent all gut microbes simultaneously, thereby also avoiding optimization-related issues with multiple microbial biomass types. These GEMs do not focus on individual microbes or their interactions, but rather on the interaction of the community with the environment or host. Such a GEM was used together with metagenomics data to study how host-microbe interactions differ in case of obesity or IBD [103]. This revealed a differential expression of enzyme groups expressed by the complete microbiota between diseased and healthy people, without investigating the roles of individual species or their interactions. The differences were found in the upregulation of membrane transport and downregulation of vitamin metabolism, nucleotide metabolism, and transcription. This study suggests that the differences in enzyme expression originate from an altered interaction between the microbes and their environment. They are the result of a change in the environment of the bacteria and do not come from a change in core metabolic processes. By combining previous approaches of modeling interspecies interactions and considering the whole microbiota as one entity, a predictive tool for dietary interventions was created [104]. The tool, CASINO—Community And Systems-level Interactive and Optimization—predicts dietary interventions based on interactions between the host, the microbiota, and the applied diet. CASINO was used to model the interactions of four microbes in two synthetic communities that differed by a single microbe. It correctly predicted the produced metabolites, including essential amino acids, and the contribution of each species to the production of each metabolite. CASINO was then used to predict the impact of a dietary intervention in 44 individuals, based on relative abundances of the most prevalent microbes in each individual before and after the intervention. The predicted production of SCFAs and amino acids mostly matched the in vivo measurements. Finally, CASINO was used to design a beneficial diet for subjects with a poor microbiota composition [104].

The use of GEMs to predict multispecies interactions and to study the influence of perturbations in environmental factors and communities is a valuable asset in microbiota function prediction. In this way, it can be predicted how individual species contribute to healthy and diseased conditions. The increase in tools for the prediction of multispecies interactions highlights the importance of this application. Moreover, these predictions were instrumental in the prediction of diets to improve the metabolic function of gut microbiota [104]. Ultimately, this research will lead to increased understanding of the interactions of the gut microbiota and its host, and on its role in gut homeostasis and (dys)function, and it will ultimately pave to way to improve human health using specific gut microbes or dietary interventions.

## Conclusion and perspectives

After a few decades of characterizing gut microbiota composition many gut microbes have been sequenced [1, 105]. Over 200 of these genome sequences have been used to generate GEMs, in most cases by automated tools [21, 28]. These GEMs have been used to predict growth phenotypes of single microbes and communities in laboratory and in vivo settings.

Here, we reviewed three ways in which GEMs contribute in elucidating gut microbiome function. We described how GEMs are used to (i) culture bacteria, (ii) predict bacterial phenotypes under changing conditions, and (iii) study the interactions both among the bacterial species and with their host.

We have shown that recent advances in automated generation of GEMs [28, 29], single-cell genomics [106], metagenomics [1, 107], and metatranscriptomics [108–110] can increase the availability and accuracy of GEMs. Metagenomics as well as single-cell genomics will yield more genome sequences of microbes that can be used for generating GEMs. Moreover, developments in single molecule sequencing will allow for closed genomes that are in the end the golden standard to be used for generating GEMs. These GEMs will contribute in understanding how both uncultured and cultured bacteria live and behave in complex ecosystems [83]. In vivo or in vitro validation of GEM predictions and subsequent GEM updates remain key in improving GEM quality and ultimately understanding the complex gut ecosystem.

GEMs allow understanding *why* species are present and *what* they do, instead of *who* they are, as was the focus in the last decades. We expect that GEMs will contribute to elucidate the mechanisms behind known probiotics, as well as in identifying new probiotics, and understanding the role of different bacteria in complex ecosystems. Ultimately, GEMs can contribute to the design of controlled interventions that steer gut composition and activity to improve human health.

## Abbreviations

FBA: Flux balance analysis
GEM: Genome-scale metabolic model
GIT: Gastrointestinal tract

## References

[1] Qin JJ, Li RQ, Raes J, Arumugam M, Burgdorf KS, Manichanh C, Nielsen T, Pons N, Levenez F, Yamada T, Mende DR, Li JH, Xu JM, Li SC, Li DF, Cao JJ, Wang B, Liang HQ, Zheng HS, Xie YL, Tap J, Lepage P, Bertalan M, Batto JM, Hansen T, Le Paslier D, Linneberg A, Nielsen HB, Pelletier E, Renault P, Sicheritz-Ponten T, Turner K, Zhu HM, Yu C, Li ST, Jian M, Zhou Y, Li YR, Zhang XQ, Li SG, Qin N, Yang HM, Wang J, Brunak S, Dore J, Guarner F, Kristiansen K, Pedersen O, Parkhill J, Weissenbach J, Bork P, Ehrlich SD, Wang J, M. Consortium (2010). A human gut microbial gene catalogue established by metagenomic sequencing. Nature.

[2] Zhou L, Foster JA (2015). Psychobiotics and the gut-brain axis: in the pursuit of happiness. Neuropsychiatr Dis Treat.

[3] Flint HJ, Scott KP, Louis P, Duncan SH (2012). The role of the gut microbiota in nutrition and health. Nat Rev Gastroenterol Hepatol.

[4] El-Semman IE, Karlsson FH, Shoaie S, Nookaew I, Soliman TH, Nielsen J (2014). Genome-scale metabolic reconstructions of Bifidobacterium adolescentis L2-32 and Faecalibacterium prausnitzii A2-165 and their interaction. BMC Syst Biol.

[5] Kelly CR, Ihunnah C, Fischer M, Khoruts A, Surawicz C, Afzali A, Aroniadis O, Barto A, Borody T, Giovanelli A, Gordon S, Gluck M, Hohmann EL, Kao D, Kao JY, McQuillen DP, Mellow M, Rank KM, Rao K, Ray A, Schwartz MA, Singh N, Stollman N, Suskind DL, Vindigni SM, Youngster I, Brandt L (2014). Fecal microbiota transplant for treatment of Clostridium difficile infection in immunocompromised patients. Am J Gastroenterol.

[6] van Nood E, Vrieze A, Nieuwdorp M, Fuentes S, Zoetendal EG, de Vos WM, Visser CE, Kuijper EJ, Bartelsman JF, Tijssen JG, Speelman P, Dijkgraaf MG, Keller JJ (2013). Duodenal infusion of donor feces for recurrent Clostridium difficile. N Engl J Med.

[7] Feist AM, Palsson BO (2008). The growing scope of applications of genome-scale metabolic reconstructions using Escherichia coli. Nat Biotechnol.

[8] Oberhardt MA, Palsson BO, Papin JA (2009). Applications of genome-scale metabolic reconstructions. Mol Syst Biol.

[9] Espey MG (2013). Role of oxygen gradients in shaping redox relationships between the human intestine and its microbiota. Free Radic Biol and Med.

[10] Ridlon JM, Kang DJ, Hylemon PB, Bajaj JS (2014). Bile acids and the gut microbiome. Curr Opin Gastroenterol.

[11] Hamer HM, Jonkers D, Venema K, Vanhoutvin S, Troost FJ, Brummer RJ (2008). Review article: the role of butyrate on colonic function. Aliment Pharmacol Ther.

[12] Smith PM, Howitt MR, Panikov N, Michaud M, Gallini CA, Bohlooly YM, Glickman JN, Garrett WS (2013). The microbial metabolites, short-chain fatty acids, regulate colonic Treg cell homeostasis. Science.

[13] Puddu A, Sanguineti R, Montecucco F, Viviani GL (2014). Evidence for the gut microbiota short-chain fatty acids as key pathophysiological molecules improving diabetes. Med Inflamm.

[14] Zoetendal EG, de Vos WM (2014). Effect of diet on the intestinal microbiota and its activity. Curr Opin Gastroenterol.

[15] Quevrain E, Maubert MA, Michon C, Chain F, Marquant R, Tailhades J, Miquel S, Carlier L, Bermudez-Humaran LG, Pigneur B, Lequin O, Kharrat P, Thomas G, Rainteau D, Aubry C, Breyner N, Afonso C, Lavielle S, Grill JP, Chassaing G, Chatel JM, Trugnan G, Xavier R, Langella P, Sokol H, Seksik P (2016). Identification of an anti-inflammatory protein from Faecalibacterium prausnitzii, a commensal bacterium deficient in Crohn’s disease. Gut.

[16] Ottman NA (2015). Host immunostimulation and substrate utilization of the gut symbiont Akkermansia muciniphila.

[17] Thiele I, Palsson BØ (2010). A protocol for generating a high-quality genome-scale metabolic reconstruction. Nat Protoc.

[18] Orth JD, Palsson B (2012). Gap-filling analysis of the i JO1366 Escherichia coli metabolic network reconstruction for discovery of metabolic functions. BMC Syst Biol.

[19] Bui TPN, Ritari J, Boeren S, de Waard P, Plugge CM, de Vos WM (2015). Production of butyrate from lysine and the Amadori product fructoselysine by a human gut commensal. Nat Comm.

[20] Kanehisa M, Goto S, Sato Y, Kawashima M, Furumichi M, Tanabe M (2014). Data, information, knowledge and principle: back to metabolism in KEGG. Nucleic Acids Res.

[21] Caspi R, Altman T, Billington R, Dreher K, Foerster H, Fulcher CA, Holland TA, Keseler IM, Kothari A, Kubo A, Krummenacker M, Latendresse M, Mueller LA, Ong Q, Paley S, Subhraveti P, Weaver DS, Weerasinghe D, Zhang PF, Karp PD (2014). The MetaCyc database of metabolic pathways and enzymes and the BioCyc collection of Pathway/Genome Databases. Nucl Acids Res.

[22] Orth JD, Palsson BØ (2010). Systematizing the generation of missing metabolic knowledge. Biotechnol Bioeng.

[23] O’Brien EJ, Monk JM, Palsson BO (2015). Using genome-scale models to predict biological capabilities. Cell.

[24] Xavier JC, Patil KR, Rocha I (2017). Integration of biomass formulations of genome-scale metabolic models with experimental data reveals universally essential cofactors in prokaryotes. Metab Eng.

[25] Orth JD, Thiele I, Palsson BØ (2010). What is flux balance analysis?. Nat Biotechnol.

[26] Kumar VS, Maranas CD (2009). GrowMatch: an automated method for reconciling in silico/in vivo growth predictions. PLoS Comput Biol.

[27] Agren R, Liu L, Shoaie S, Vongsangnak W, Nookaew I, Nielsen J (2013). The RAVEN toolbox and its use for generating a genome-scale metabolic model for Penicillium chrysogenum. PLoS Comput Biol.

[28] Henry CS, DeJongh M, Best AA, Frybarger PM, Linsay B, Stevens RL (2010). High-throughput generation, optimization and analysis of genome-scale metabolic models. Nat Biotechnol.

[29] Magnusdottir S, Heinken A, Kutt L, Ravcheev DA, Bauer E, Noronha A, Greenhalgh K, Jager C, Baginska J, Wilmes P, Fleming RM, Thiele I (2017). Generation of genome-scale metabolic reconstructions for 773 members of the human gut microbiota. Nat Biotechnol.

[30] Rajilic-Stojanovic M, de Vos WM (2014). The first 1000 cultured species of the human gastrointestinal microbiota. FEMS Microbiol Rev.

[31] Lagier JC, Khelaifia S, Alou MT, Ndongo S, Dione N, Hugon P, Caputo A, Cadoret F, Traore SI, Seck EH, Dubourg G, Durand G, Mourembou G, Guilhot E, Togo A, Bellali S, Bachar D, Cassir N, Bittar F, Delerce J, Mailhe M, Ricaboni D, Bilen M, Dangui Nieko NP, Dia Badiane NM, Valles C, Mouelhi D, Diop K, Million M, Musso D, Abrahao J, Azhar EI, Bibi F, Yasir M, Diallo A, Sokhna C, Djossou F, Vitton V, Robert C, Rolain JM, La Scola B, Fournier PE, Levasseur A, Raoult D (2016). Culture of previously uncultured members of the human gut microbiota by culturomics. Nat Microbiol.

[32] Abdallah RA, Beye M, Diop A, Bakour S, Raoult D, Fournier PE. The impact of culturomics on taxonomy in clinical microbiology. Ant Van Leeuwenh. 2017. 10.1007/s10482-017-0871-1 28389704

[33] Ritari J, Salojarvi J, Lahti L, de Vos WM (2015). Improved taxonomic assignment of human intestinal 16S rRNA sequences by a dedicated reference database. BMC Genomics.

[34] Fodor AA, DeSantis TZ, Wylie KM, Badger JH, Ye Y, Hepburn T, Hu P, Sodergren E, Liolios K, Huot-Creasy H, Birren BW, Earl AM (2012). The “most wanted” taxa from the human microbiome for whole genome sequencing. PLoS One.

[35] Konikoff T, Gophna U (2016). Oscillospira: a central, enigmatic component of the human gut microbiota. Trends Microbiol.

[36] Mackie RI, Aminov RI, Hu W, Klieve AV, Ouwerkerk D, Sundset MA, Kamagata Y (2003). Ecology of uncultivated Oscillospira species in the rumen of cattle, sheep, and reindeer as assessed by microscopy and molecular approaches. Appl Environ Microbiol.

[37] Cuiv PO, Smith WJ, Pottenger S, Burman S, Shanahan ER, Morrison M (2015). Isolation of genetically tractable most-wanted bacteria by metaparental mating. Sci Rep.

[38] Teusink B, van Enckevort FHJ, Francke C, Wiersma A, Wegkamp A, Smid EJ, Siezen RJ (2005). In silico reconstruction of the metabolic pathways of Lactobacillus plantarum: comparing predictions of nutrient requirements with those from growth experiments. Appl Environ Microbiol.

[39] Teusink B, Smid EJ (2006). Modelling strategies for the industrial exploitation of lactic acid bacteria. Nat Rev Microbiol.

[40] Teusink B, Wiersma A, Molenaar D, Francke C, de Vos WM, Siezen RJ, Smid EJ (2006). Analysis of growth of Lactobacillus plantarum WCFS1 on a complex medium using a genome-scale metabolic model. J Biol Chem.

[41] Wegkamp A, Teusink B, De Vos W, Smid E (2010). Development of a minimal growth medium for Lactobacillus plantarum. LAM.

[42] Saulnier DM, Santos F, Roos S, Mistretta T-A, Spinler JK, Molenaar D, Teusink B, Versalovic J (2011). Exploring metabolic pathway reconstruction and genome-wide expression profiling in Lactobacillus reuteri to define functional probiotic features. PLoS One.

[43] dos Santos FB, de Vos WM, Teusink B (2013). Towards metagenome-scale models for industrial applications—the case of Lactic Acid Bacteria. Curr Opin Biotechnol.

[44] Kleerebezem M, Boekhorst J, van Kranenburg R, Molenaar D, Kuipers OP, Leer R, Tarchini R, Peters SA, Sandbrink HM, Fiers MW (2003). Complete genome sequence of Lactobacillus plantarum WCFS1. Proc Natl Acad Sci U S A.

[45] Oliveira AP, Nielsen J, Förster J (2005). Modeling Lactococcus lactis using a genome-scale flux model. BMC Microbiol.

[46] Aller K, Adamberg K, Timarova V, Seiman A, Feštšenko D, Vilu R (2014). Nutritional requirements and media development for Lactococcus lactis IL1403. Appl Microbiol Biotechnol.

[47] Becker SA, Palsson BØ (2005). Genome-scale reconstruction of the metabolic network in Staphylococcus aureus N315: an initial draft to the two-dimensional annotation. BMC Microbiol.

[48] Heinemann M, Kümmel A, Ruinatscha R, Panke S (2005). In silico genome‐scale reconstruction and validation of the Staphylococcus aureus metabolic network. Biotechnol Bioeng.

[49] Zarecki R, Oberhardt MA, Reshef L, Gophna U, Ruppin E (2014). A novel nutritional predictor links microbial fastidiousness with lowered ubiquity, growth rate, and cooperativeness. PLoS Comput Biol.

[50] Heinken A, Khan MT, Paglia G, Rodionov DA, Harmsen HJ, Thiele I (2014). Functional metabolic map of Faecalibacterium prausnitzii, a beneficial human gut microbe. J Bacteriol.

[51] Bauer E, Laczny CC, Magnusdottir S, Wilmes P, Thiele I (2015). Phenotypic differentiation of gastrointestinal microbes is reflected in their encoded metabolic repertoires. Microbiome.

[52] Nielsen HB, Almeida M, Juncker AS, Rasmussen S, Li JH, Sunagawa S, Plichta DR, Gautier L, Pedersen AG, Le Chatelier E, Pelletier E, Bonde I, Nielsen T, Manichanh C, Arumugam M, Batto JM, Dos Santos MBQ, Blom N, Borruel N, Burgdorf KS, Boumezbeur F, Casellas F, Dore J, Dworzynski P, Guarner F, Hansen T, Hildebrand F, Kaas RS, Kennedy S, Kristiansen K, Kultima JR, Leonard P, Levenez F, Lund O, Moumen B, Le Paslier D, Pons N, Pedersen O, Prifti E, Qin JJ, Raes J, Sorensen S, Tap J, Tims S, Ussery DW, Yamada T, Renault P, Sicheritz-Ponten T, Bork P, Wang J, Brunak S, Ehrlich SD, M. Consortium (2014). Identification and assembly of genomes and genetic elements in complex metagenomic samples without using reference genomes. Nat Biotechnol.

[53] Lasken RS (2012). Genomic sequencing of uncultured microorganisms from single cells. Nat Rev Microbiol.

[54] Kolinko S, Richter M, Glockner FO, Brachmann A, Schuler D. Single-cell genomics of uncultivated deep-branching magnetotactic bacteria reveals a conserved set of magnetosome genes. Environ Microbiol. 2016;18(1):21–37. 10.1111/1462-2920.12907 26060021

[55] Rath CM, Alexandrov T, Higginbottom SK, Song J, Milla ME, Fischbach MA, Sonnenburg JL, Dorrestein PC (2012). Molecular analysis of model gut microbiotas by imaging mass spectrometry and nanodesorption electrospray ionization reveals dietary metabolite transformations. Anal Chem.

[56] Lewis NE, Nagarajan H, Palsson BO (2012). Constraining the metabolic genotype-phenotype relationship using a phylogeny of in silico methods. Nat Rev Microbiol.

[57] Oberhardt MA, Puchalka J, Fryer KE, Martins dos Santos VA, Papin JA (2008). Genome-scale metabolic network analysis of the opportunistic pathogen Pseudomonas aeruginosa PAO1. J Bacteriol.

[58] Ventura M, O’Flaherty S, Claesson MJ, Turroni F, Klaenhammer TR, van Sinderen D, O’Toole PW (2009). Genome-scale analyses of health-promoting bacteria: probiogenomics. Nat Rev Microbiol.

[59] Lewis NE, Hixson KK, Conrad TM, Lerman JA, Charusanti P, Polpitiya AD, Adkins JN, Schramm G, Purvine SO, Lopez-Ferrer D, Weitz KK, Eils R, Konig R, Smith RD, Palsson BO (2010). Omic data from evolved E. coli are consistent with computed optimal growth from genome-scale models. Mol Syst Biol.

[60] Guo CJ, Chang FY, Wyche TP, Backus KM, Acker TM, Funabashi M, Taketani M, Donia MS, Nayfach S, Pollard KS, Craik CS, Cravatt BF, Clardy J, Voigt CA, Fischbach MA (2017). Discovery of reactive microbiota-derived metabolites that inhibit host proteases. Cell.

[61] Plovier H, Everard A, Druart C, Depommier C, Van Hul M, Geurts L, Chilloux J, Ottman N, Duparc T, Lichtenstein L, Myridakis A, Delzenne NM, Klievink J, Bhattacharjee A, van der Ark KC, Aalvink S, Martinez LO, Dumas ME, Maiter D, Loumaye A, Hermans MP, Thissen JP, Belzer C, de Vos WM, Cani PD (2017). A purified membrane protein from Akkermansia muciniphila or the pasteurized bacterium improves metabolism in obese and diabetic mice. Nat Med.

[62] Steeb B, Claudi B, Burton NA, Tienz P, Schmidt A, Farhan H, Maze A, Bumann D (2013). Parallel exploitation of diverse host nutrients enhances Salmonella virulence. PLoS Pathog.

[63] Bartell JA, Yen P, Varga JJ, Goldberg JB, Papin JA (2014). Comparative metabolic systems analysis of pathogenic Burkholderia. J Bacteriol.

[64] Ramsey DM, Wozniak DJ (2005). Understanding the control of Pseudomonas aeruginosa alginate synthesis and the prospects for management of chronic infections in cystic fibrosis. Mol Microbiol.

[65] Großeholz R, Koh C-C, Veith N, Fiedler T, Strauss M, Olivier B, Collins BC, Schubert OT, Bergmann F, Kreikemeyer B, Aebersold R, Kummer U (2016). Integrating highly quantitative proteomics and genome-scale metabolic modeling to study pH adaptation in the human pathogen Enterococcus faecalis. NPJ Syst Biol Appl.

[66] Hoppe A (2012). What mRNA abundances can tell us about metabolism. Metabolites.

[67] Rocca JD, Hall EK, Lennon JT, Evans SE, Waldrop MP, Cotner JB, Nemergut DR, Graham EB, Wallenstein MD (2015). Relationships between protein-encoding gene abundance and corresponding process are commonly assumed yet rarely observed. ISME J.

[68] Weaver DS, Keseler IM, Mackie A, Paulsen IT, Karp PD (2014). A genome-scale metabolic flux model of Escherichia coli K-12 derived from the EcoCyc database. BMC Syst Biol.

[69] Zhang SW, Gou WL, Li Y (2017). Prediction of metabolic fluxes from gene expression data with Huber penalty convex optimization function. Mol Biosyst.

[70] King ZA, Drager A, Ebrahim A, Sonnenschein N, Lewis NE, Palsson BO (2015). Escher: a web application for building, sharing, and embedding data-rich visualizations of biological pathways. PLoS Comput Biol.

[71] Machado D, Herrgård M (2014). Systematic evaluation of methods for integration of transcriptomic data into constraint-based models of metabolism. PLoS Comput Biol.

[72] Kim J, Reed JL (2014). Refining metabolic models and accounting for regulatory effects. Curr Opin Biotechnol.

[73] Faria JP, Overbeek R, Xia FF, Rocha M, Rocha I, Henry CS (2014). Genome-scale bacterial transcriptional regulatory networks: reconstruction and integrated analysis with metabolic models. Brief Bioinform.

[74] Chandrasekaran S, Price ND (2013). Metabolic constraint-based refinement of transcriptional regulatory networks. Plos Comp Biol.

[75] Carrera J, Estrela R, Luo J, Rai N, Tsoukalas A, Tagkopoulos I (2014). An integrative, multi-scale, genome-wide model reveals the phenotypic landscape of Escherichia coli. Mol Syst Biol.

[76] Karr JR, Sanghvi JC, Macklin DN, Gutschow MV, Jacobs JM, Bolival B, Assad-Garcia N, Glass JI, Covert MW (2012). A whole-cell computational model predicts phenotype from genotype. Cell.

[77] Kim MK, Lun DS (2014). Methods for integration of transcriptomic data in genome-scale metabolic models. Comput Struct Biotechnol J.

[78] Chandrasekaran S, Price ND (2010). Probabilistic integrative modeling of genome-scale metabolic and regulatory networks in Escherichia coli and Mycobacterium tuberculosis. Proc Natl Acad Sci U S A.

[79] Novichkov PS, Kazakov AE, Ravcheev DA, Leyn SA, Kovaleva GY, Sutormin RA, Kazanov MD, Riehl W, Arkin AP, Dubchak I, Rodionov DA (2013). RegPrecise 3.0-A resource for genome-scale exploration of transcriptional regulation in bacteria. BMC Genomics.

[80] McCloskey D, Palsson BØ, Feist AM (2013). Basic and applied uses of genome‐scale metabolic network reconstructions of Escherichia coli. Mol Syst Biol.

[81] Klitgord N, Segre D (2010). Environments that induce synthetic microbial ecosystems. Plos Comp Biol.

[82] Freilich S, Zarecki R, Eilam O, Segal ES, Henry CS, Kupiec M, Gophna U, Sharan R, Ruppin E (2011). Competitive and cooperative metabolic interactions in bacterial communities. Nat Commun.

[83] Ji B, Nielsen J (2015). From next-generation sequencing to systematic modeling of the gut microbiome. Front Genet.

[84] Borenstein E (2012). Computational systems biology and in silico modeling of the human microbiome. Brief Bioinform.

[85] Tzamali E, Poirazi P, Tollis IG, Reczko M (2011). A computational exploration of bacterial metabolic diversity identifying metabolic interactions and growth-efficient strain communities. BMC Syst Biol.

[86] Salimi F, Zhuang K, Mahadevan R (2010). Genome-scale metabolic modeling of a clostridial co-culture for consolidated bioprocessing. Biotechnol J.

[87] Sun J, Haveman SA, Bui O, Fahland TR, Lovley DR (2010). Constraint-based modeling analysis of the metabolism of two Pelobacter species. BMC Syst Biol.

[88] Levy R, Borenstein E (2013). Metabolic modeling of species interaction in the human microbiome elucidates community-level assembly rules. Proc Natl Acad Sci U S A.

[89] Stolyar S, Van Dien S, Hillesland KL, Pinel N, Lie TJ, Leigh JA, Stahl DA (2007). Metabolic modeling of a mutualistic microbial community. Mol Syst Biol.

[90] Feng X, Xu Y, Chen Y, Tang YJ (2012). MicrobesFlux: a web platform for drafting metabolic models from the KEGG database. BMC Syst Biol.

[91] Zelezniak A, Andrejev S, Ponomarova O, Mende DR, Bork P, Patil KR (2015). Metabolic dependencies drive species co-occurrence in diverse microbial communities. Proc Natl Acad Sci U S A.

[92] Harcombe WR, Riehl WJ, Dukovski I, Granger BR, Betts A, Lang AH, Bonilla G, Kar A, Leiby N, Mehta P, Marx CJ, Segre D (2014). Metabolic resource allocation in individual microbes determines ecosystem interactions and spatial dynamics. Cell Rep.

[93] Mahadevan R, Edwards JS, Doyle FJ (2002). Dynamic flux balance analysis of diauxic growth in Escherichia coli. Biophys J.

[94] Harcombe W (2010). Novel cooperation experimentally evolved between species. Evolution.

[95] Chubiz LM, Granger BR, Segre D, Harcombe WR (2015). Species interactions differ in their genetic robustness. Front Microbiol.

[96] Bauer E, Zimmermann J, Baldini F, Thiele I, Kaleta C (2017). BacArena: individual-based metabolic modeling of heterogeneous microbes in complex communities. PLoS Comput Biol.

[97] Becker N, Kunath J, Loh G, Blaut M (2011). Human intestinal microbiota: characterization of a simplified and stable gnotobiotic rat model. Gut Microbes.

[98] Flint HJ, Duncan SH, Louis P (2017). The impact of nutrition on intestinal bacterial communities. Curr Opin Microbiol.

[99] Sigurdsson MI, Jamshidi N, Steingrimsson E, Thiele I, Palsson BØ (2010). A detailed genome-wide reconstruction of mouse metabolism based on human Recon 1. BMC Syst Biol.

[100] Thiele I, Swainston N, Fleming RM, Hoppe A, Sahoo S, Aurich MK, Haraldsdottir H, Mo ML, Rolfsson O, Stobbe MD (2013). A community-driven global reconstruction of human metabolism. Nat Biotechno.

[101] Browne HP, Forster SC, Anonye BO, Kumar N, Neville BA, Stares MD, Goulding D, Lawley TD (2016). Culturing of ‘unculturable’ human microbiota reveals novel taxa and extensive sporulation. Nature.

[102] Hasler R, Sheibani-Tezerji R, Sinha A, Barann M, Rehman A, Esser D, Aden K, Knecht C, Brandt B, Nikolaus S, Schauble S, Kaleta C, Franke A, Fretter C, Muller W, Hutt MT, Krawczak M, Schreiber S, Rosenstiel P (2016). Uncoupling of mucosal gene regulation, mRNA splicing and adherent microbiota signatures in inflammatory bowel disease. Gut.

[103] Greenblum S, Turnbaugh PJ, Borenstein E (2012). Metagenomic systems biology of the human gut microbiome reveals topological shifts associated with obesity and inflammatory bowel disease. Proc Natl Acad Sci U S A.

[104] Shoaie S, Ghaffari P, Kovatcheva-Datchary P, Mardinoglu A, Sen P, Pujos-Guillot E, de Wouters T, Juste C, Rizkalla S, Chilloux J, Hoyles L, Nicholson JK, Dore J, Dumas ME, Clement K, Backhed F, Nielsen J, Consortium M-O (2015). Quantifying diet-induced metabolic changes of the human gut microbiome. Cell Metab.

[105] Peterson J, Garges S, Giovanni M, McInnes P, Wang L, Schloss JA, Bonazzi V, McEwen JE, Wetterstrand KA, Deal C, Baker CC, Di Francesco V, Howcroft TK, Karp RW, Lunsford RD, Wellington CR, Belachew T, Wright M, Giblin C, David H, Mills M, Salomon R, Mullins C, Akolkar B, Begg L, Davis C, Grandison L, Humble M, Khalsa J, Little AR, Peavy H, Pontzer C, Portnoy M, Sayre MH, Starke-Reed P, Zakhari S, Read J, Watson B, Guyer M, N.H.W. Grp (2009). The NIH human microbiome project. Genome Res.

[106] Blainey PC (2013). The future is now: single-cell genomics of bacteria and archaea. FEMS Microbiol Rev.

[107] Gill SR, Pop M, DeBoy RT, Eckburg PB, Turnbaugh PJ, Samuel BS, Gordon JI, Relman DA, Fraser-Liggett CM, Nelson KE (2006). Metagenomic analysis of the human distal gut microbiome. Science.

[108] Bailly J, Fraissinet-Tachet L, Verner MC, Debaud JC, Lemaire M, Wesolowski-Louvel M, Marmeisse R (2007). Soil eukaryotic functional diversity, a metatranscriptomic approach. ISME J.

[109] Baldrian P, Lopez-Mondejar R (2014). Microbial genomics, transcriptomics and proteomics: new discoveries in decomposition research using complementary methods. Appl Microbiol Biotechnol y.

[110] Maurice CF, Haiser HJ, Turnbaugh PJ (2013). Xenobiotics shape the physiology and gene expression of the active human gut microbiome. Cell.

